# Endogenous CCL21-Ser deficiency reduces B16–F10 melanoma growth by enhanced antitumor immunity

**DOI:** 10.1016/j.heliyon.2023.e19215

**Published:** 2023-08-19

**Authors:** Ryonosuke Fujie, Kaoru Kurowarabe, Yuki Yamada, Kakeru Fujiwara, Hayato Nakatani, Kenta Tsutsumi, Ryota Hayashi, Hinami Kawahata, Megumi Miyamoto, Madoka Ozawa, Tomoya Katakai, Yousuke Takahama, Izumi Ohigashi, Haruko Hayasaka

**Affiliations:** a Department of Science, Graduate School of Science and Engineering, Kindai University, 3-4-1, Kowakae, Higashiosaka, Osaka 577-8502, Japan; b Faculty of Science & Engineering, Kindai University, 3-4-1, Kowakae, Higashiosaka, Osaka 577-8502, Japan; c Department of Immunology, Niigata University Graduate School of Medical and Dental Sciences, 1-757 Asahimachi-dori, Chuo-ku, Niigata 951-8510, Japan; d Division of Experimental Immunology, Institute of Advanced Medical Sciences, University of Tokushima, 3-18-15 Kuramoto, Tokushima 770-8503, Japan; e Thymus Biology Section, Experimental Immunology Branch, National Cancer Institute, National Institutes of Health, Bethesda, MD 20892, United States; f Research Institute for Science and Technology, Kindai University, 3-4-1, Kowakae, Higashiosaka, Osaka 577-8502, Japan

**Keywords:** Melanoma, Chemokine, Lymph node, CD8, Treg

## Abstract

The chemokine CCL21 regulates immune and cancer cell migration through its receptor CCR7. The *Ccl21a* gene encodes the isoform CCL21-Ser, predominantly expressed in the thymic medulla and the secondary lymphoid tissues. This study examined the roles of CCL21-Ser in the antitumor immune response in *Ccl21a*-knockout (KO) mice. The *Ccl21a-*KO mice showed significantly decreased growth of B16–F10 and YUMM1.7 melanomas and increased growth of MC38 colon cancer, despite no significant difference in LLC lung cancer and EO771 breast cancer. The B16–F10 tumor in *Ccl21a*-KO mice showed melanoma-specific activated CD8^+^ T cell and NK cell infiltration and higher Treg counts than wild-type mice. B16–F10 tumors in *Ccl21a*-KO mice showed a reduction in the positive correlation between the ratio of regulatory T cells (Tregs) to activated CD8^+^ T cells and tumor weight. In *Ccl21a*-KO tumor, the intratumoral Tregs showed lower co-inhibitory receptors TIM-3 and TIGIT. Taken together, these results suggest that endogenous CCL21-Ser supports melanoma growth *in vivo* by maintaining Treg function and suppressing antitumor immunity by CD8^+^ T cells.

## Highlights

• The host-derived CCL21-Ser has different effects on tumor growth depending on tumor cell types.
• Lack of the host-derived CCL21-Ser reduces B16–F10 melanoma growth.
• *Ccl21a*-KO melanomas are frequently infiltrated with activated CD8^+^ T cells.
• Tregs in *Ccl21a*-KO melanomas show decreased levels of co-inhibitory receptors.
• The host-derived CCL21-Ser regulates Treg-mediated anti-melanoma immunity.

## Abbreviations

***LN***: Lymph node
***WT***: Wild-type
***KO***: Knockout
***Treg***: Regulatory T cells
***mAbs***: monoclonal antibodies
***ILNs***: Inguinal lymph nodes
***dLNs***: draining lymph nodes

## 1 Introduction

The homeostatic chemokine CCL21 has two isoforms: CCL21-Ser, encoded by the *Ccl21a* gene, with a serine at the 65th position, and CCL21-Leu, encoded by multiple genes, including *Ccl21b*, with leucine at that position [1,2]. CCL21-Ser is expressed in the stromal cells of secondary lymphoid tissues and thymic epithelial cells [[2], [3], [4]], whereas CCL21-Leu is expressed in the lymphatic endothelial cells of peripheral tissues [1,4]. Both isoforms transmit intracellular signals by binding to the chemokine receptor CCR7, expressed on thymocytes, naïve lymphocytes, central memory T cells, regulatory T cells, and mature dendritic cells [5]. Knockout (KO) studies in mice have demonstrated the critical roles of CCL21 in immune cell migration and tissue localization. *Ccl21a-*KO mice develop autoimmune disease-like symptoms due to an incomplete negative selection of autoreactive T cells during lymphocyte differentiation in the thymus [3]. The *Ccr7*-KO mice showed attenuated thymocyte migration (from the cortex to the medulla), reduced T cell and dendritic cell localization in the secondary lymphoid tissues [[6], [7], [8]]. The dysregulation of immune response in those gene-KO mice indicates that CCR7 signaling is essential for proper immune cell regulation *in vivo*. Growing evidence suggests that the interaction between CCL21 and CCR7 in the tumor microenvironment is associated with poor prognosis in various types of human cancer. CCL21 produced by lymphatic endothelial cells generates a concentration gradient that induces migration of cancer cells to the lymph nodes (LNs) in a CCR7-dependent manner [9,10]. Consistent with these findings, clinical studies in breast cancer, melanoma, gastric cancer, esophageal squamous epithelial cells carcinoma, and chronic lymphocytic leukemia, have demonstrated that CCR7 expression is correlated with cancer cell metastasis to LNs [[11], [12], [13]]. Thus, CCL21 expressed by cancer cells and stromal cells in the tumor microenvironment confers metastatic capacity to cancer cells to the regional LNs.

The ability of CCL21 to stimulate T cell and DC migration suggests that CCL21 plays a key role in an effective immune response in the tumor microenvironment. A significant number of studies have demonstrated that systemic or local CCL21 expression promotes anti-tumor response by recruiting lymphocytes and DCs into tumor tissue [14]. The expression of CCL21 in cancer cells including melanoma, liver cancer, and prostate cancer cells, induces immune responses and inhibits tumor growth [[15], [16], [17]]. CCL21-Ser in melanoma and lung carcinoma cells suppressed tumor growth by developing blood vessels similar to the high endothelial venules in LNs, leading to an enhanced antitumor immunity by mobilizing naïve lymphocytes to the tumor tissue [18]. In addition, CCL21 promotes antitumor immune response by inducing tumor antigen presentation and suppressing the production of immunosuppressive cytokines in human breast cancer cells [19]. Intratumoral administration of DCs expressing CCL21 in mice induced anti-tumor immunity and markedly suppressed the growth of subcutaneous lung cancer, suggesting the benefit of CCL21 expression in DC-based cancer immunotherapy [20]. In contrast, in another study, CCL21-Ser produced by murine melanoma cells induced peritumoral LN-like structures and recruited immunosuppressive cells, enhancing the melanoma growth [21]. Thus, although the impact of the CCL21/CCR7 signaling on anti-tumor immunity remains controversial, the fact that CCL21 expression can induce an immune response provides a rationale for the use of CCL21 in cancer immunotherapy. For optimal therapeutic use of CCL21, the contribution of host-derived CCL21-Ser to tumor growth and its role *in vivo* must be fully understood. Therefore, this study assessed the role of endogenous CCL21-Ser in tumor growth and immune cell infiltration in tumor tissues using *Ccl21a-*KO mice.

## 2 Material and methods

### 2.1 Cells

The mouse melanoma B16–F10 and YUMM1.7 were obtained from the American Type Culture Collection. The mouse Lewis lung carcinoma (LLC) and colon adenocarcinoma MC38 cell lines were kindly provided by Drs. K. Moriwaki of Osaka Medical Collage and CM. Lee of Osaka University, respectively. Breast cancer EO771 cell line was obtained from CH3 BioSystems (Amherst, NY, USA). YUMM1.7 were cultured in Dulbecco's Modified Eagle medium/Ham's F-12 (FUJIFILM Wako Pure Chemicals, Osaka, Japan) supplemented with 10% (v/v) fetal calf serum. Other cell lines were cultured in Dulbecco's Modified Eagle medium (FUJIFILM Wako Pure Chemicals, Osaka, Japan) supplemented with 10% (v/v) fetal calf serum.

### 2.2 Animals

C57BL/6 N and B6 Albino (B6N-*Tyr*^*c-Brd*^/BrdCrCrl) were purchased from Nihon SLC (Hamamatsu, Japan) and Charles River Laboratories Japan, Inc. (Yokohama, Japan), respectively. The original *Ccl21a-KO* mice, CDB1030K http://www.clst.riken.jp/arg/mutant%20mice%20list.html [3], were backcrossed three times onto the C57BL/6 or B6 Albino backgrounds before continuing with sibling mating. The mice were euthanized by a lethal dose of isoflurane via inhalation. For *in vivo* tumor formation, B16–F10, LLC and MC38 cells (1 × 10^6^ per mouse) were subcutaneously injected into 6-12-week-old mice of either sex and YUMM1.7 cells (1 × 10^5^ per mouse) were injected into 6-week-old female mice with 27-G needles. EO771 cells (5 × 10^5^ per mouse) were injected into the fifth right mammary fat pad of female mice. The volume of mammary tumor was determined by caliper measurements and calculated using formula V = width^2^ × length × 0.5*.* All experiments were conducted in accordance with the approved guidelines from Kindai University. The experimental protocols for use of laboratory animals were approved by the Ethics Review Committee of Kindai University (KASE-2021-001).

### 2.3 In vivo bioluminescence imaging

The B16–F10 cells expressing the click beetle luciferase gene (CBRluc) were obtained by transfection with the CBRluc-containing plasmid pCR3.1-Uni (Invitrogen, Carlsbad, CA, USA) followed by selection with 1 mg/ml G418 (FUJIFILM Wako Pure Chemicals). Wild-type (WT) or *Ccl21a-*KO mice with C57BL/B6 Albino backgrounds were subcutaneously injected on the back with the luciferase-expressing B16–F10 cells. The mice were anesthetized with isoflurane, injected intraperitoneally with 3 mg (150 mg/kg) D-luciferin (VivoGlo, Promega), and imaged using an IVIS Imaging System (Caliper Life Sciences, Hopkinton, MA, USA).

### 2.4 RT-PCR analysis

Total RNA was purified using the RNeasy Mini Kit (QIAGEN), and cDNA was synthesized using the FastGene Scriptase Ⅱ cDNA 5x Ready Mix (NIPPON Genetics Co., Ltd.). PCR was performed with Gene RED PCR Mix Plus (NIPPON Gene Co., Ltd.). The primer sequences used are described below:

*Ccl21a* forward: 5′-aactcaaccacaatcatg-3’
*Ccl21a* reverse: 5′-cttgaagttcgtggggga-3’
*gapdh* forward: 5′-ggaaagctgtggcgttggcgtgat-3’
*gapdh* reverse: 5′-ctgttgctgtagccgtattc-3’

### 2.5 Immunohistochemistry

Fresh-frozen tumor specimens dissected two weeks after cell injection were subjected to immunohistochemical staining. Frozen sections of 10-μm thickness were fixed with ice-cold acetone, incubated with 50% Immunoblock (DS Pharma) in PBS for 30 min, and were treated with APC-conjugated anti-B220, Alexa Fluor 594-conjugated anti-CD3, and rabbit anti-mouse CCL21 antibodies listed on Table 1 overnight at 4 °C. CCL21 was detected by a tyramide signal amplification system (Thermo Fisher Scientific Inc.). Tissues were treated with horseradish peroxidase (HRP)-conjugated donkey anti-goat IgG for 30 min at room temperature, followed by Alexa Fluor 488 tyramide reagent at 1:800 dilution (B40953, Thermo Fisher Scientific Inc.) for 10 min at room temperature. The tissues were stained with 2 μg/ml Hoechst 33342 (Invitrogen) for 10 min, mounted with Fluoromount-G (SouthernBiotech), and observed with a confocal laser scanning microscope (FV1200-D, Olympus).

**Table 1:** List of reagent and resource used in this study.

Reagent or Resource	Source	Clone	Concentration (μg/ml)	RRID
FITC anti-mouse CD3ε	BioLegend	145-2C11	5	AB_312671
Alexa Fluor 594 anti-mouse CD3ε	BioLegend	17A2	5	AB_2563427
PE anti-mouse CD3ε	BioLegend	145-2C11	2	AB_312673
PE/Cy7 anti-mouse CD45	BioLegend	30-F11	1	AB_312979
APC anti-mouse B220	eBioscience	RA3-6B2	5	AB_469395
APC anti-mouse CD8α	BioLegend	53–6.7	2	AB_312751
Biotin anti-mouse CD8α	BioLegend	53–6.7	2	AB_312743
FITC anti-mouse CD4	BioLegend	RM4-5	2	AB_312713
PE anti-mouse CD4	BioLegend	GK1.5	1.25	AB_312693
APC anti-mouse CD4	BioLegend	RM4-5	2	AB_312719
PE anti-human/mouse CD44	BioLegend	IM7	1	AB_312959
FITC anti-mouse CD62L	BioLegend	MEL-14	2	AB_313093
PE anti-mouse CD25	BioLegend	3C7	2.5	AB_312847
PerCP/Cy5.5 anti-mouse CD127	BioLegend	A7R34	2	AB_1937273
Biotin anti-mouse CD127	Thermo Fisher Scientific	A7R34	2	AB_466589
Alexa Fluor 488 anti-mouse FoxP3	BioLegend	MF-14	2	AB_1089114
PE anti-mouse CCR7	BioLegend	4B12	2	AB_389357
APC anti-mouse F4/80	BioLegend	BM8	2	AB_893481
FITC anti-mouse MHC class II	BioLegend	M5/114.15.2	1	AB_313321
Biotin anti-mouse CD11c	eBioscience	N418	2.5	AB_466363
Alexa Fluor 647 anti-mouse CD69	BioLegend	H1.2F3	2	AB_492848
FITC anti-mouse NK1.1	BioLegend	PK136	2.5	AB_313392
PE anti-mouse Ki-67	BioLegend	11F6	2.5	AB_2716014
anti-mouse PD-1	BioLegend	RMP1-30	2	AB_313422
anti-mouse Tim-3	BioLegend	RMT3-23	1	AB_2561655
anti-mouse TIGIT	BioLegend	1G9	1	AB_10960139
APC anti-mouse IFN-γ	BioLegend	XMG1.2	2	AB_315404
anti-mouse CCL21	R&D	AF457	1	AB_2072083
HRP donkey anti-goat IgG	Abcam	polyclonal	1:400	AB_955421
DyLight 649 Streptavidin	Vector Laboratories	N/A	1	N/A
PE-conjugated Streptavidin	BioLegend	N/A	1	N/A
H-2Kb TRP-2 tetramer-PE	MBL International	N/A	10 μL/sample	N/A

### 2.6 Flow cytometry

The tumor tissues were enzymatically treated at 37 °C for 1.5 h with RPMI 1640 medium containing 0.1% bovine serum albumin, 1 mg/ml collagenase type Ι (9001-12-1, Wako) and 2 μg/ml deoxyribonuclease I (9003-98-9, Sigma). They were subsequently treated with 0.83% NH_4_Cl and 0.17 M Tris-Cl (pH 7.65) for erythrocyte hemolysis. LN cells were treated with HB-197 cell culture supernatant containing an anti-CD16/32 (Fc-γ receptor) antibody. The cell suspension was treated with antibodies and fluorescent reagents listed on Table 1. The cell number was counted with Flow-count Fluorospheres (Beckman Coulter). The data analysis and interpretation were performed on a BD LSRFortessa system and the FlowJo software (BD Biosciences).

### 2.7 *In vivo* depletion of CD8^+^ T cells using mAb treatment

Six-week-old *Ccl21a-*KO mice were injected intraperitoneally with 100 μg of GoInVivo purified anti-mouse CD8 mAb (clone 53–6.7, BioLegend) or allogeneic isotype immunoglobulin (control Ig) three days prior to B16–F10 injection. Antibody injection was repeated on the day of B16–F10 cell injection and on day 7 post injection. Spleens and tumors were harvested from each mouse on day 14 and the CD8 population was examined by flow cytometry.

### 2.8 Analysis of the effector activity of *in vitro* cultured splenocytes

A detailed description of the protocol is presented previously [22]. Briefly, splenocytes of tumor-bearing or control mice were cultured with or without MMC-treated B16–F10 cells in culture media containing 100 U/ml recombinant mouse IL-2 (BioLegend). To assess effector activity of CD8^+^ T cells by target cell viability, CD8^+^ T cells co-cultured with or without B16–F10 cells were added to B16–F10 or LLC cells expressing firefly luciferase. Bioluminescence was measured by real-time luminometer (Kronos Dio, Atto).

### 2.9 Statistics

Data were evaluated by F-test for equal variances, then analyzed by Student's *t*-test for equal variances, and by Welch's *t*-test or Mann-Whitney *U* test for unequal variances using StatPlus software version v7 (AnalystSoft Inc.).

## 3 Results

### 3.1 Lack of host-derived CCL21-Ser reduces B16–F10 melanoma growth

**Fig. 1:**
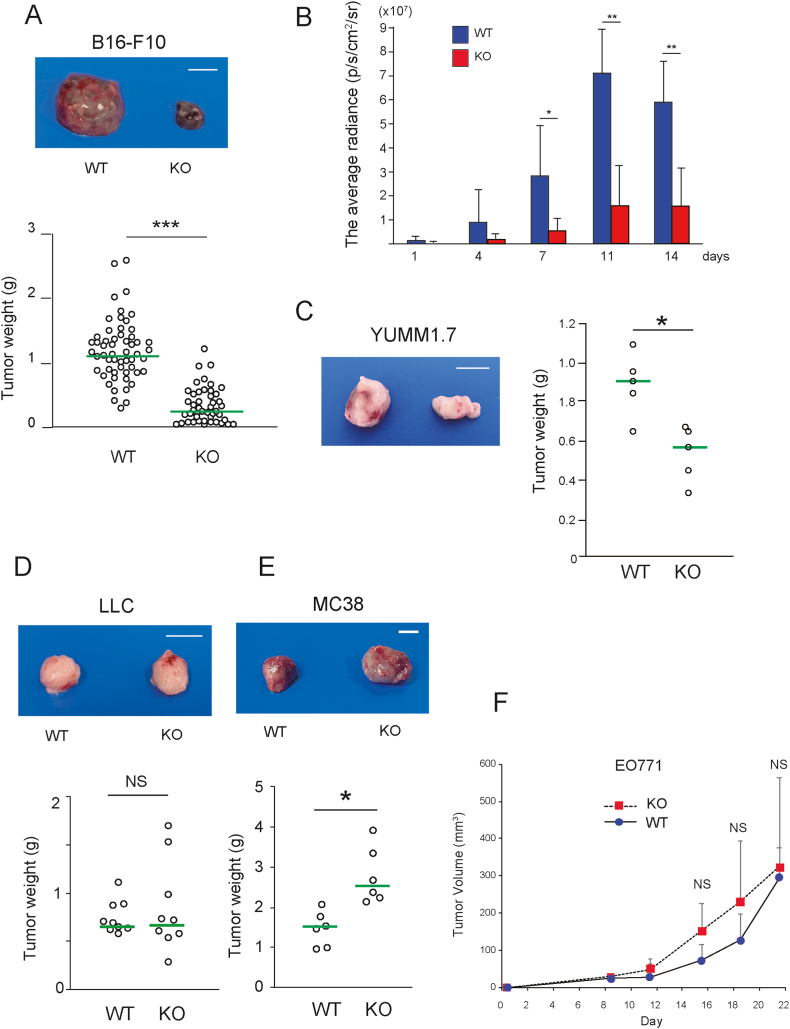
**The effect of *Ccl21a* deficiency on melanoma growth *in vivo*.** (A) Representative images of B16–F10-derived tumors formed in the wild-type (WT) or *Ccl21a*-KO mice (left). Scale bar: 10 mm. B16–F10-derived tumors formed in WT (*n* = 55) or KO (*n* = 45) were weighed two weeks after injection (right). Each dot represents a sample of the individual mouse, and green lines show the median. Mann-Whitney's *U* test was used to compare differences between the two groups. ***, *P* < 0.001, NS: Not Significant. (B) B16–F10 cells stably expressing click beetle luciferase were subcutaneously injected into WT or *Ccl21a*-KO on the B6 Albino background (*n* = 6). An IVIS Lumina system at the indicated time points measured luminescence counts. The average radiance of IVIS-imaged mice (p/s/cm^2^/sr) was shown. Welch's *t*-test was used to compare the differences between the two groups. *, *P* < 0.05, **, *P* < 0.005 (C) The effect of *Ccl21a* expression on YUMM1.7, (D) LLC carcinoma, and (E) MC38 colon carcinoma growth in WT and Ccl21a-KO mice. Tumors derived from YUMM1.7 (WT; *n* = 5, KO; *n* = 5) and MC38 (WT; *n* = 6, KO; *n* = 6) were weighed three-weeks after injection. Tumors derived from LLC (WT; *n* = 9, KO; *n* = 9) were weighed two-weeks after injection. (F) EO771 tumor volume was measured using a vernier caliper (WT; *n* = 5, KO; *n* = 5). Mann-Whitney's *U* test was used as the significance test. NS: Not Significant.

The B16–F10-derived melanoma in the *Ccl21a-*KO mice had significantly lower tumor weight than that of the wild-type (WT) mice (Fig. 1A). *In vivo* bioluminescence imaging showed B16–F10 growth in WT mice until day 11 (Fig. 1B), whereas the luminescence in *Ccl21a-*KO mice was already lower than that in WT by day 4, suggesting that tumor growth was suppressed relatively at the early phase in the *Ccl21a*-KO mice. Similarly, the growth of YUMM1.7 melanoma was significantly lower in *Ccl21a*-KO mice, whereas not significantly different in LLC lung cancer and EO771 breast cancer, and significantly increased in MC38 colon cancer (Fig. 1C–F). Therefore, the reduction in tumor growth in *Ccl21a*-KO mice is likely to be a melanoma-selective phenomenon. The expression of *Ccl21a* and CCR7 are undetectable in B16–F10 cells cultured *in vitro* and those cells in tumor tissues (Supplementary Fig. 1A and B). Immunohistochemistry detected no ectopic lymphoid structures with B and T cell aggregates in tumors of WT and *Ccl21a*-KO mice (Supplementary Fig. 1C). Moreover, CCL21-Ser expression was undetectable in tumor tissues, suggesting the contribution of host-derived CCL21-Ser and CCR7 on nontumor cells to B16–F10 tumor growth.

### 3.2 *Ccl21a*-KO melanomas are frequently infiltrated with activated CD8^+^ T cells

**Fig. 2:**
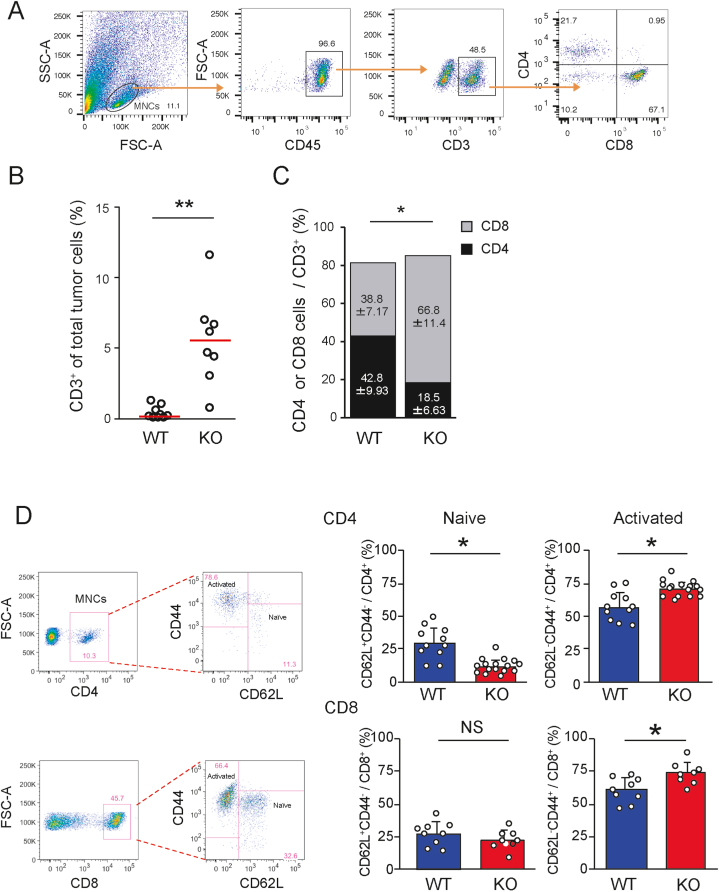
**Analysis of tumor-infiltrated T cell subsets in wild-type and *Ccl21a*-deficient mice.** The B16–F10-derived tumor generated in WT and *Ccl21a*-KO mice were harvested two weeks after injection. The tumor-infiltrated T cell subset was analyzed by flow cytometry. (A) The lymphocyte subset was distinguished by the forward and side scatter profiles, and the proportion of CD8^+^ or CD4^+^ cells in the CD3^+^ cell population was analyzed. (B) The percentage (*n* = 9 in WT and *n* = 8 in KO) of CD3^+^ cells in the tumor. Each dot represents an individual sample. The data were analyzed by Mann-Whitney *U* test (**, *P* < 0.01). Red lines represent the median. (C) The percentage of CD8^+^ or CD4^+^ cells in the CD3^+^ cell fraction (*n* = 6). The data are shown as mean ± SD analyzed by Student's *t-*test (*, *P* < 0.05). (D) The percentage of naïve (CD44^−^ CD62L^+^) or activated (CD44^+^ CD62L^−^) T cells in the CD4^+^ (WT: *n* = 11, KO: *n* = 16) and the CD8^+^ cell subsets (WT: *n* = 9, KO: *n* = 8) were analyzed. The data are shown as mean ± SD analyzed by Student's *t*-test (*, *P* < 0.05, NS: Not Significant).

**Fig. 3:**
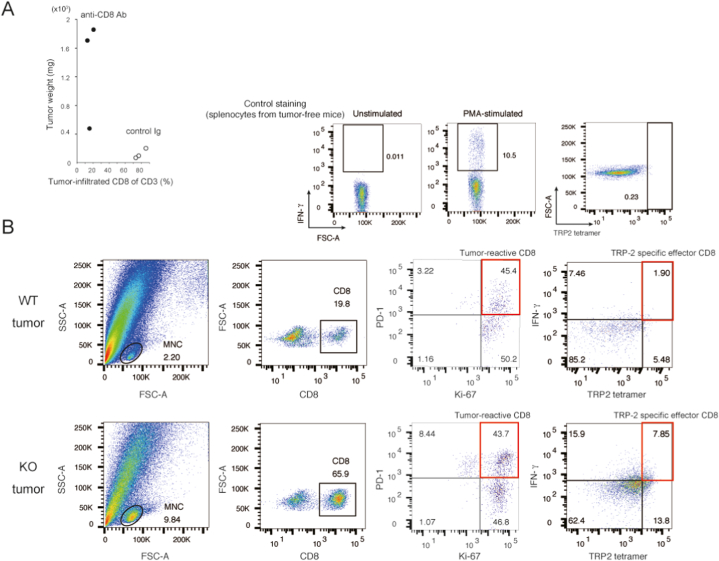
**The effect of intratumoral CD8**^**+**^**T cells on melanoma growth.** (A) The effect of CD8^+^ T cell depletion on tumor weight in the *Ccl21a*-KO mice. Anti-CD8 antibody or control immunoglobulin (control Ig) was administered to the *Ccl21a*-KO mice (*n* = 3) three days before, on days 0 and 7 of transplantation. The percentage of CD8^+^ cells and tumor weight in each mouse two weeks after transplantation was measured. (B) The expression of tumor-reactive T cell marker molecules (PD-1, Ki-67, IFN-γ, TRP2 tetramer) in the intratumoral CD8^+^ T cells. Results are representative of two biological repeats.

**Fig. 4:**
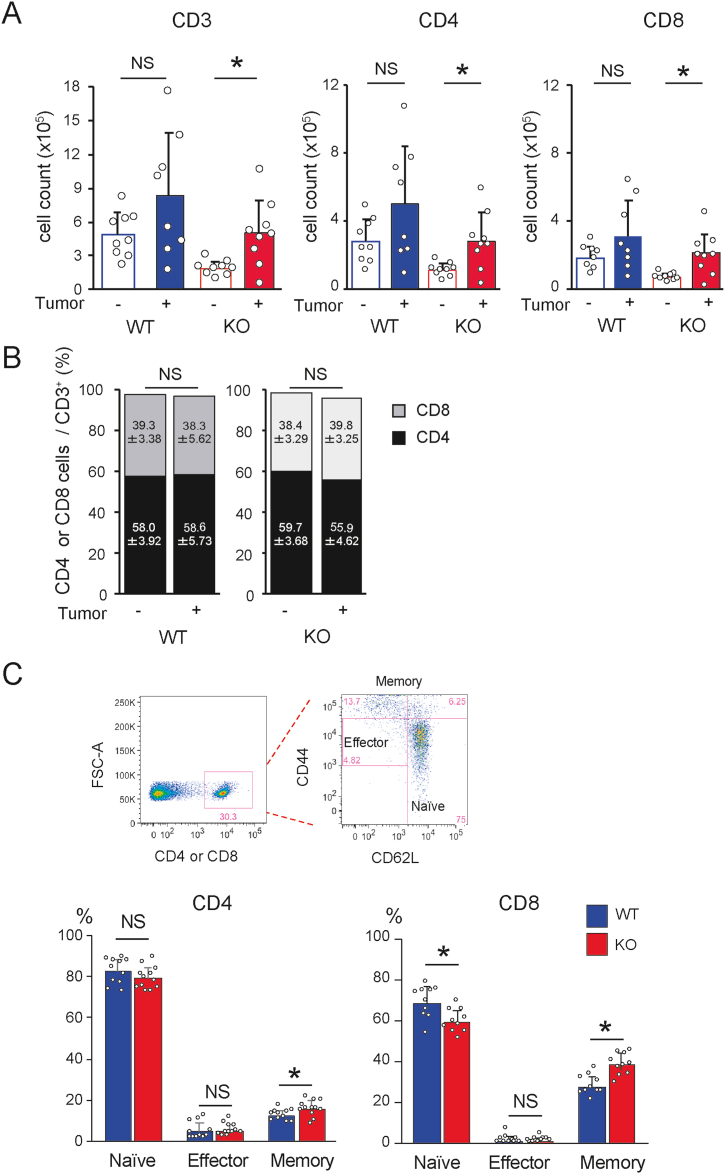
**Changes in T cell subsets in the tumor-draining LNs of *Ccl21a*-KO mice.** The number and percentage of T cells in the tumor-draining inguinal LNs of tumor-bearing or control mice were analyzed by flow cytometry. (A) The number of CD3^+^, CD4^+^, and CD8^+^ cells in the inguinal LNs of control or tumor-bearing mice (*n* = 9). (B) The percentage of CD4^+^ or CD8^+^ T cells in the CD3^+^ cell subset. (C) Naïve (CD62L^high^CD44^lo^), effector (CD62L^lo^CD44^mid^), and effector memory (CD62L^lo^CD44^high^, Tem) T cell percentage of CD4^+^ and CD8^+^ T cells in WT and *Ccl21a*-KO mice of tumor-bearing mice (*n* = 10). The data are shown as mean ± SD analyzed by Student's *t-*test (*, *P* < 0.05, NS: Not Significant).

To decipher the mechanisms underlying decreased B16–F10 tumorigenicity in *Ccl21a-*KO mice, we hypothesized that a stronger antitumor immunity was induced in the KO mice. A flow cytometric analysis of CD3^+^, CD4^+^, and CD8^+^ T cell populations within the intratumoral cells (Fig. 2A) revealed that the proportion of tumor infiltrating T cells, especially CD8^+^ T cells, was significantly higher in the KO mice than in WT (Fig. 2B and C). Additionally, a significant increase in activated T cells and a decrease in naïve T cells was observed in the KO mice (Fig. 2D). The depletion of CD8^+^ T cells by anti-CD8 mAb enhanced tumor growth in *Ccl21a*-KO tumors, indicating that the decrease in B16–F10 growth in *Ccl21a-*KO mice is CD8^+^ T cell-dependent (Fig. 3A). The frequency of CD8^+^ T cells expressing tumor-reactive T cell markers, PD-1, and Ki-67, was comparable between *Ccl21a*-KO and WT mice; however, the frequency of H-2Kb TRP-2 tetramer-specific IFN-γ^+^ CD8^+^ T cells was elevated in *Ccl21a*-KO tumors, suggesting that anti-melanoma immunity was enhanced in the KO mice (Fig. 3B). The frequency of NK cells was also significantly increased in *Ccl21a*-KO tumor compared with WT, suggesting a possible contribution of NK cells to the decreased B16–F10 growth in *Ccl21a-*KO mice (Supplementary figure 2). In contrast, the frequencies of macrophage and DC infiltration were comparable between *Ccl21a-*KO and WT mice. We hypothesized that the KO mice have an elevated tumor-specific immune response in draining LNs (dLNs). We observed no significant difference in CD4^+^ or CD8^+^ T cell counts before and after tumor formation in WT dLNs, whereas they were significantly increased in *Ccl21a*-KO dLNs (Fig. 4A). Although the CD4/CD8 ratio of tumor-draining and non-dLNs was comparable (Fig. 4B), the frequencies of naïve T cells were decreased and those of effector memory T cells were increased in *Ccl21a*-KO dLNs (Fig. 4C).

**Fig. 5:**
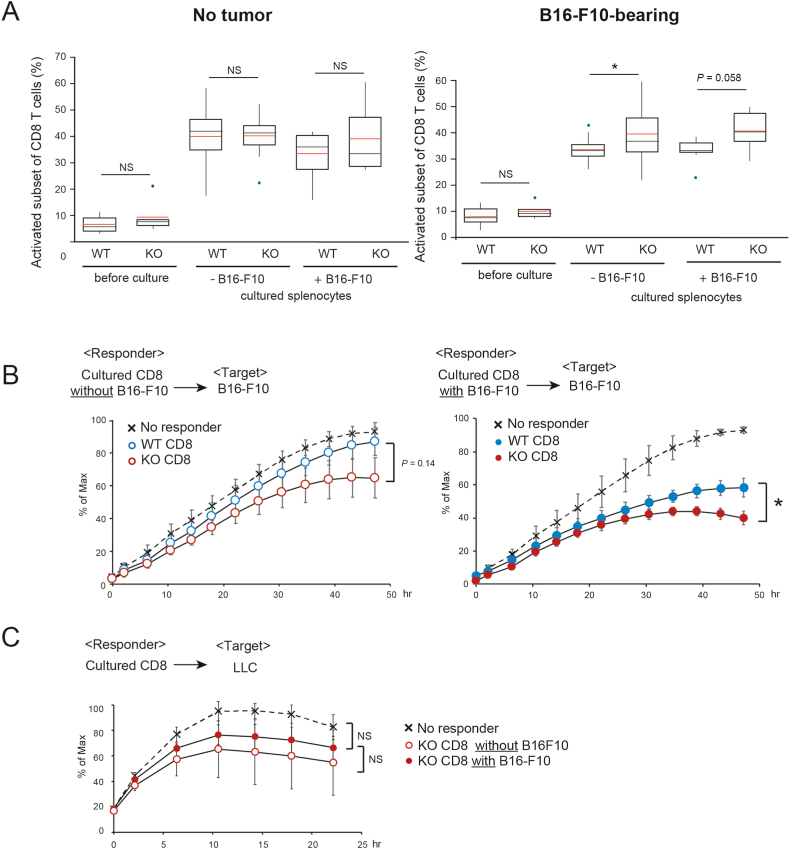
**The effect of CD8**^**+**^**T cells in B16–F10-bearing *Ccl21a*-KO mice on *in vitro* melanoma growth.** Splenocytes prepared form no tumor control or B16–F10-bearing wild-type or *Ccl21a*-KO mice two weeks after B16–F10 transplantation were cocultured with or without B16–F10 cells for three days. (A) The frequencies of activated CD8^+^ T cells (CD62L^−^CD44^mid^ and CD62L^−^CD44^hi^) with B16–F10 tumor (*n* = 12, 22, 20, 18, 8, 8) or without B16–F10 tumor (*n* = 11, 6, 20, 15, 8, 6) were analyzed by flow cytometry. Statistical significance was defined by Mann-Whitney *U* test. NS: Not Significant *: *P* < 0.05 (B) Effector activity of spleen-derived CD8^+^ T cells was evaluated by the bioluminescence derived from the target B16–F10 cell viability. The luminescence values is expressed as 100% of the maximum value when responder cells are not added. *: *P* < 0.05 (C) Bystander cytotoxicity with KO-derived CD8^+^ T cells was analyzed using LLC as target cells (*n* = 3).

We assessed the effector function of CD8^+^ T cells in tumor-bearing *Ccl21a*-KO and WT mice. To this end, we cultured CD8^+^ T cells derived from tumor-bearing mice and monitored the time course of target B16–F10 cell viability using a bioluminescence-based method [22]. The tumor-bearing splenocytes from *Ccl21a*-KO mice showed a higher frequency of activated CD8 cells and a higher impact on B16–F10 cell viability than WT (Fig. 5A and B, respectively). They did not exert a bystander effect on LLC cells (Fig. 5C), suggesting that more B16–F10-specific effector T cells were generated in *Ccl21a*-KO mice.

### 3.3 *Ccl21a*-KO melanomas are infiltrated with Tregs with decreased levels of co-inhibitory receptors

**Fig. 6:**
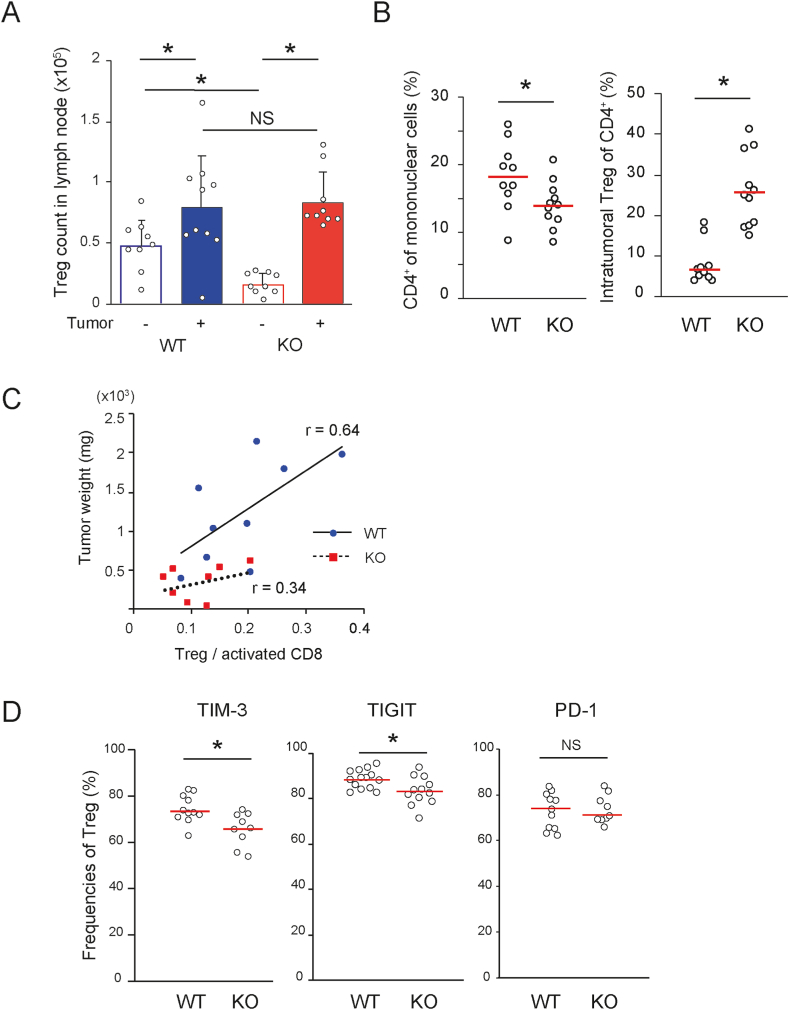
**Tregs in the tumor-draining LNs and B16–F10-derived tumors of WT and *Ccl21a*-KO mice.** The number and percentage of tumor-infiltrated Tregs in B16–F10-derived tumors in WT and *Ccl21a*-KO mice were analyzed by flow cytometry. Each plot represents an individual sample. (A) The number of Tregs in the tumor-draining inguinal LNs of control (n = 9) or tumor-bearing mice (WT: n = 10, KO: n = 9, mean ± SD). The data are shown as mean ± SD analyzed by Student's *t*-test (*, *P* < 0.05, NS: Not Significant). Each dot represents an individual sample. (B) The percentage of CD4^+^ lymphocyte subset (left) and tumor-infiltrated Tregs of CD4^+^ cells (right) in tumor-bearing WT and *Ccl21a*-KO mice (WT: n = 10, KO: n = 11). The data are shown as Mann-Whitney *U* test (*, P < 0.05, NS: Not Significant). Red lines represent the median. (C) A scatter plot of mouse tumor weight versus intratumoral Treg/activated CD8 ratio (WT: *n* = 9, KO: *n* = 8). (D) The percentage of PD-1^+^ (WT: n = 11, KO: n = 9), Tim-3^+^ (WT: *n* = 11, KO: *n* = 9), and TIGIT^+^ (WT: *n* = 14, KO: *n* = 12) of tumor-infiltrated Tregs. The data are shown as Mann-Whitney *U* test (*, *P* < 0.05, NS: Not Significant).

Regulatory T cells (Tregs) regulate the differentiation of naïve CD8^+^ T cells into memory T cells and suppress melanoma growth [23,24]. Although the number of CD4^+^CD25^+^CD127^lo^ cells in *Ccl21a*-KO dLNs was significantly lower than that of WT dLNs without tumors (Supplementary Fig. 3A, Fig. 6A), the number increased more significantly in *Ccl21a*-KO dLNs (approximately 6-fold) compared with the WT dLNs (approximately 1.7-fold) as the tumor grew. The majority of CD4^+^CD25^+^CD127^lo^ cells in the tumors were Tregs, as approximately 80% of them express FoxP3 (Supplementary Fig. 3B). Although the proportion of CD4^+^ T cells was lower in *Ccl21a*-KO than WT tumors, the Treg frequency of CD4^+^ T cells was higher in *Ccl21a*-KO (Fig. 6B), suggesting that Treg accumulation was more efficiently induced in *Ccl21a*-KO tumor.

In contrast to the higher Treg frequency, the *Ccl21a*-KO tumor showed an apparent reduction in the positive correlation between the ratio of Tregs to activated CD8^+^ T cells and tumor weight (Fig. 6C). Therefore, we hypothesized that the immune suppressive function is impaired in *Ccl21a*-KO mice, regardless of their efficient recruitment to the tumor tissues. To understand the functional abnormalities in *Ccl21a*-KO intratumoral Tregs, we examined the expression of co-inhibitory receptors TIM-3 and TIGIT, which are associated with Treg function in the tumor microenvironment [25,26]. The frequencies of TIM-3^+^ and TIGIT^+^ Tregs were reduced in *Ccl21a*-KO compared with control mice (Fig. 6D), consistent with the idea that *Ccl21a*-KO Tregs are functionally impaired. The frequency of myeloid-derived suppressor cells (MDSCs), immune cells from the myeloid lineage with immunosuppressive phenotypes, was not significantly different between *Ccl21a*-KO and wild-type tumor (Supplementary Fig. 2B), suggesting that the MDSCs do not significantly contribute to the decreased tumor growth in *Ccl21a*-KO mice.

## 4 Discussion

Our results shed light on the function of host-derived CCL21 in tumor growth *in vivo.* We have demonstrated for the first time that B16–F10, a poorly immunogenic cell line widely used in the studies of tumor-induced T cell anergy [24], efficiently elicited immune response in the absence of host-derived CCL21-Ser. B16–F10 tumors formed in *Ccl21a*-KO mice showed higher T cell ratios and increased the numbers of activated CD4^+^, CD8^+^ T cells, and NK cells. In addition, the activated CD8^+^ T cells recognizing TRP-2 were significantly increased in *Ccl21a*-KO tumors than in WT tumors, suggesting that host-derived CCL21-Ser has a suppressive effect on anti-melanoma immunity.

The results using a mouse melanoma model are consistent with cohort studies showing that melanoma patients with high CCL21 expression have shorter overall survival (the Human Protein Atlas, https://www.proteinatlas.org/ENSG00000137077-CCL21/pathology/melanoma). Among the five cell lines used, tumor growth of B16–F10 and YUMM1.7 was decreased in *Ccl21a*-KO mice, whereas that of LLC and EO771 was unchanged, and that of MC38 was increased, suggesting that the effect of CCL21-Ser deficiency is selective for melanoma. We speculate that differences in immunogenicity between B16–F10 and MC38 may have caused opposite effects in these cell lines. B16–F10 and MC38 are a poorly immunogenic and an immunogenic cell line, respectively [27]. In MC38 tumors, CD3^+^ and Treg frequencies were unchanged in *CCL21a*-KO mice, whereas the CD8/CD4 ratio was increased (Supplementary figure 4A). Notably, the frequency of macrophages was significantly reduced in *CCL21a*-KO mice compared with WT mice (Supplementary figure 2B and 4B). A pervious paper has demonstrated that M1 macrophages, well-characterized activated macrophages with antitumorigenic properties, inhibit MC38 tumor growth [28] and they express CCR7 and migrate toward a CCL21 gradient [29]. Since CCL21 was detectable in MC38 tumors by immunohistochemical analysis (Supplementary figure 4C), it is possible that the tumor growth of MC38 in *CCL21a*-KO mice is due to decreased infiltration of M1 macrophages by CCL21-Ser deficiency, resulting in a reduced anti-tumor immune response. Thus, differences in tumor microenvironment and immunogenicity may account for the different effects of *Ccl21a* deficiency. Our study does not clarify whether CCL21-Ser supports melanoma growth only in certain genetic backgrounds. To understand this point, it is necessary to analyze allogeneic transplants of various cancer cells in animals with different genetic backgrounds.

Given that activated CD8^+^ T cells and NK cells were more efficiently infiltrated in *Ccl21a*-KO melanoma compared with WT counterpart, these cytotoxic immune cell population primarily contribute to suppression of melanoma growth. CD8^+^ T and NK cells are major effector cells against various type of tumors and their anti-tumor effects are increased by Treg depletion [[30], [31], [32]]. In our study, however, the increased activated CD8^+^ T cells was not associated with a numerical decrease in Tregs in *Ccl21a*-KO mice. The ratio of Treg to activated CD8^+^ T cells did not positively correlate with tumor weight, we therefore speculated that Tregs in *Ccl21a*-KO tumors have reduced immunosuppressive activity. In line with this idea, we examined the expression of TIM-3 and TIGIT, co-inhibitory molecules which are crucial for Treg function [26,33], and exhibited that TIM-3^+^ and TIGIT^+^ Treg frequencies were reduced in *Ccl21a*-KO tumors. In *Ccl21a*-KO tumors, both TIM-3^+^ and TIGIT^+^ Tregs were reduced by less than 10%, however, since the two molecules have been reported to act synergistically [18], the simultaneous reduction of TIM-3 and TIGIT may have resulted in a significant decrease in Treg suppressive activity. Unfortunately, it is technically impossible to directly analyze the intratumoral Treg activity, because the number of intratumoral Tregs is approximately 1∼5 × 10^3^ (0.1% of total KO tumor sample), which is far less than the number required for *in vitro* suppression assay. Further experiments with high sensitivity are needed to confirm intratumoral Treg dysfunction in *Ccl21a*-KO tumors.

The *Ccr7*-KO mice showed increased infiltration of inflamed tissues with inactive Tregs, suggesting that the CCL21-Ser/CCR7 axis is critical for functional Treg development *in vivo* [34]. Our findings are consistent with this idea and indicate that endogenous CCL21-Ser expression is essential for the development of functional Tregs in antitumor immunity. Since the predominant Treg subset in human and mouse tumors is the thymus-derived naturally occurring Treg [35], a lack of CCL21-Ser expression in the *Ccl21a*-KO thymus may cause intrinsic dysfunction of naturally occurring Tregs and accelerated CD8^+^ T-mediated tumor rejection. However, we speculate that this is unlikely, as our preliminary data do not demonstrate dysfunction of *Ccl21a*-KO splenic Tregs on effector CD4^+^ T cell proliferation *in vitro*. Alternatively, the ineffective suppressive function of *Ccl21a*-KO Tregs is possibly by their defective LN positioning, as demonstrated in *Ccr7*-KO Tregs [34].

In mouse melanoma models, multiple studies have demonstrated that depletion of Tregs by anti-CD25 antibody enhances antitumor immune responses [23,36]. However, a clinical trial of transient Treg depletion in melanoma patients with anti-CD25 antibodies did not improve the efficacy of the DC vaccine due to a concomitant depletion of effector T cells [37]. Although further studies are needed on the molecular mechanisms leading to Treg dysfunction in *Ccl21a*-KO mice with B16–F10, following our idea that the reduced Treg recruitment in LNs is responsible for tumor regression in *Ccl21a*-KO mice, inactivating Treg function by blocking Treg entry into tumor-dLNs could be a new approach to effective immunotherapy.

## Author contributions

Haruko Hayasaka: Conceived and designed the experiments; Performed the experiments; Analyzed and interpreted the data; Wrote the paper.

Ryonosuke Fujie; Kaoru Kurowarabe; Yuki Yamada; Kakeru Fujiwara; Hayato Nakatani; Kenta Tsutsumi; Ryota Hayashi; Hinami Kawahata; Megumi Miyamoto; Madoka Ozawa: Performed the experiments; Analyzed and interpreted the data.

Tomoya Katakai; Yousuke Takahama; Izumi Ohigashi: Contributed reagents, materials, analysis tools or data; Analyzed and interpreted the data.

## Data availability statement

Data will be made available on request.

## Additional information

The initial draft prior to formal peer review has been deposited onto Research Square preprint platform (https://doi.org/10.21203/rs.3.rs-1500142/v1).

## Declaration of competing interest

The authors declare the following financial interests/personal relationships which may be considered as potential competing interests: Haruko Hayasaka reports financial support was provided by the Ministry of Education, Culture, Sports, Science and Technology, Japan.
