# Supplementary material for: Endogenous CCL21-Ser deficiency reduces B16–F10 melanoma growth by enhanced antitumor immunity
Source: Heliyon. 2023 Aug 19;9(8):e19215. doi: 10.1016/j.heliyon.2023.e19215 (PMC10469598; doi:10.1016/j.heliyon.2023.e19215)
Supplement: Multimedia component 1 [file mmc1.pdf]

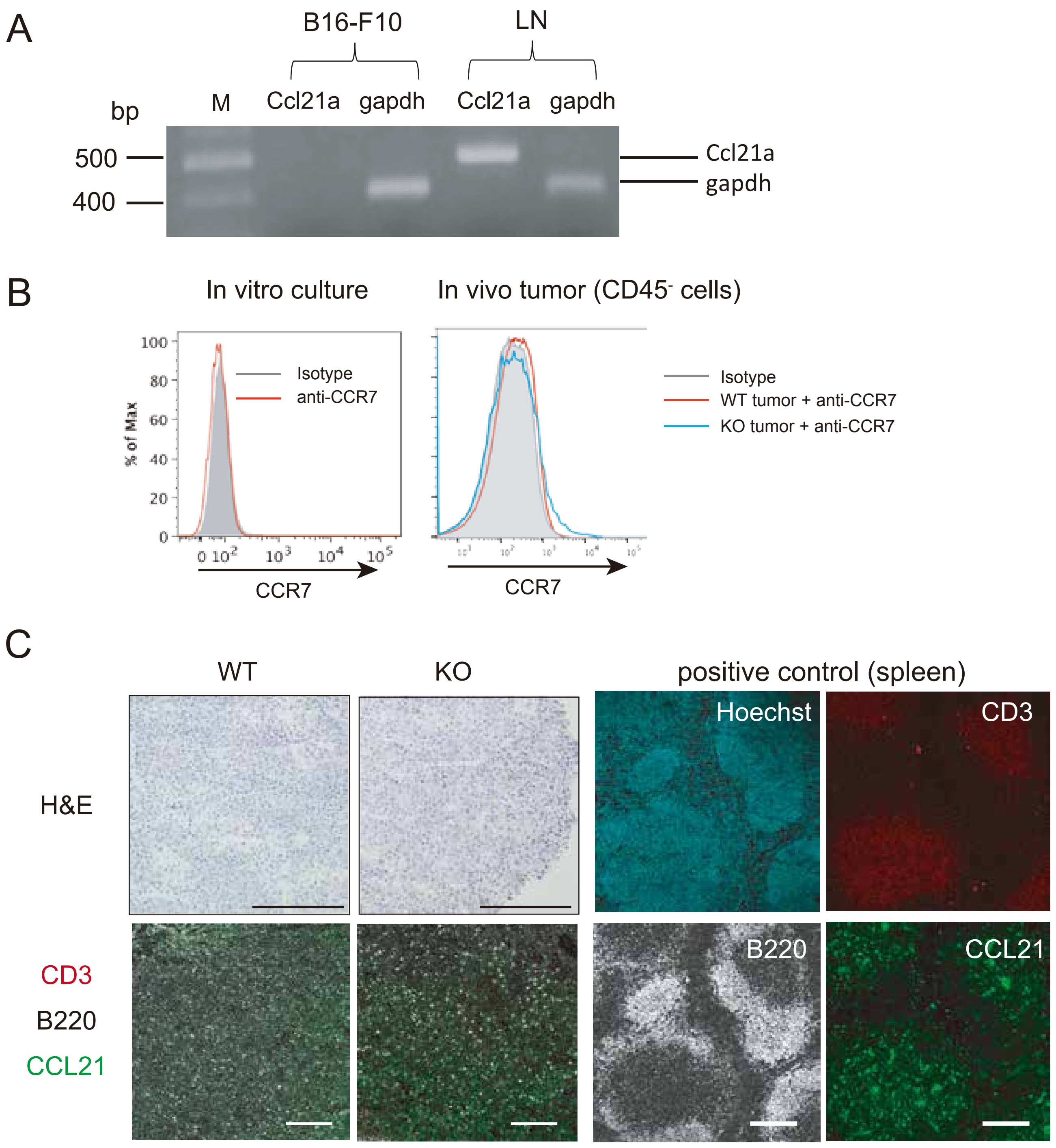

Supplementary Figure 1 The expression of CCL21 and CCR7 in B16-F10 cells.

(A) *Ccl21a* and the internal contol *gapdh* expression in cultured B16-F10 cells were detected by RT-PCR. The amplified products of *Ccl21a* and *gapdh* transcripts are approximately 500-bp and 400-bp, respctively. LN: Lymph node

(B) The CCR7 expression levels of B16-F10 cells cultured in vitro (left) and in tumor tissue (right) were analyzed by flow cytometry with an anti-mouse CCR7 monoclonal antibody. Results are a representative of two biological repeats

(C) Hematoxylin & Eosin staining and Immunohistochemical analysis of B16-F10 tumor tissue in WT and KO mice. Results are a representative of three biological repeats.Scale bar: 200 μm

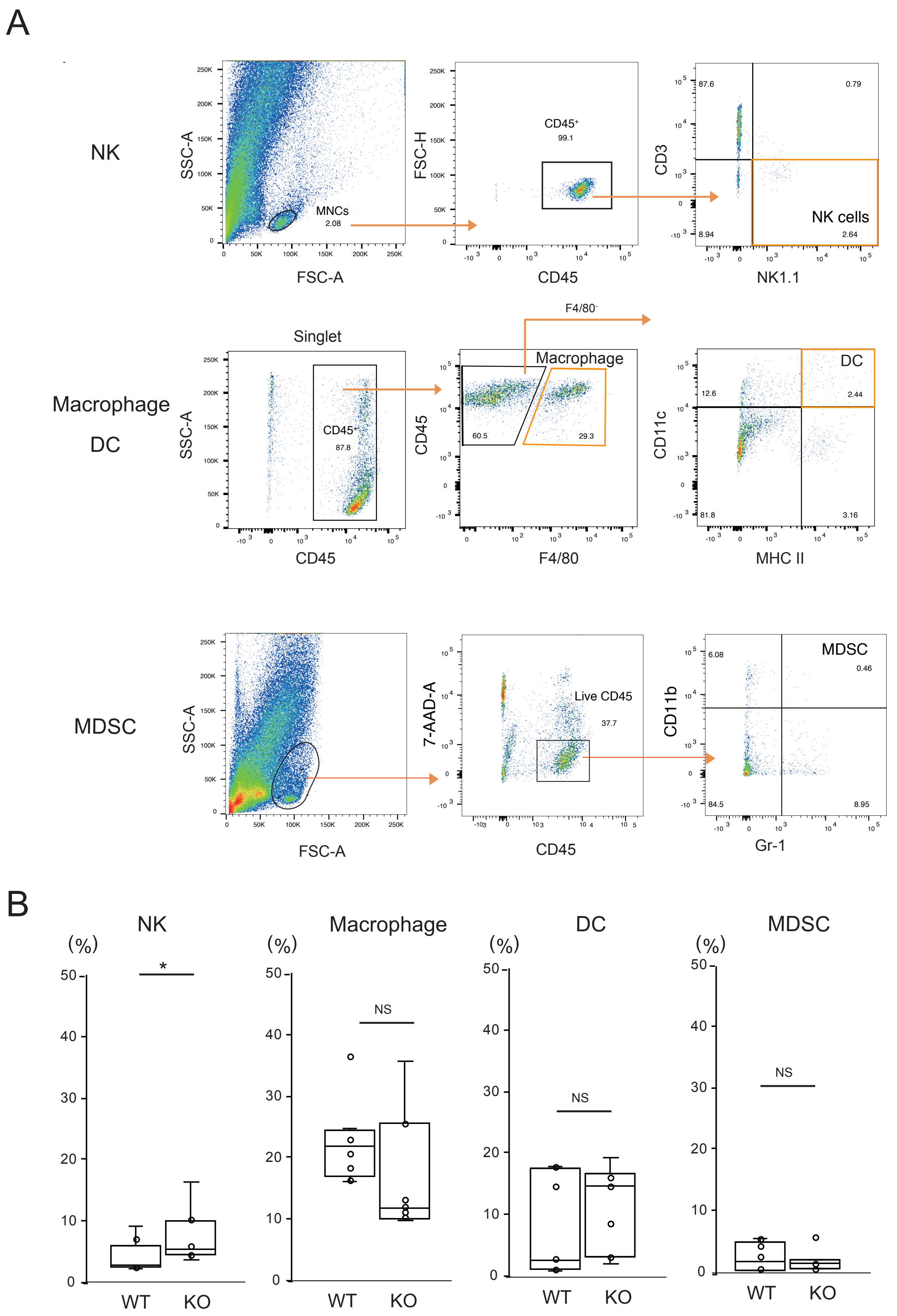

Supplementary Figure 2 Immune cell infiltration in B16-F10 tumors. (A) Representative gating strategies of NK cells (NK1.1^{+} CD3^{-} CD69^{+}), macrophage (F4/80^{+}), DCs (F4/80^{-} MHC class II^{+} CD11c^{+}) and MDSCs (Gr-1^{+} CD11b^{+}) in B16-F10 tumors. MNCs: Mononuclear cells. (B) The percentages of immune cell subsets of CD45^{+} cells in B16-F10 tumors in wild-type (WT) or Ccl21a-KO mice (KO).

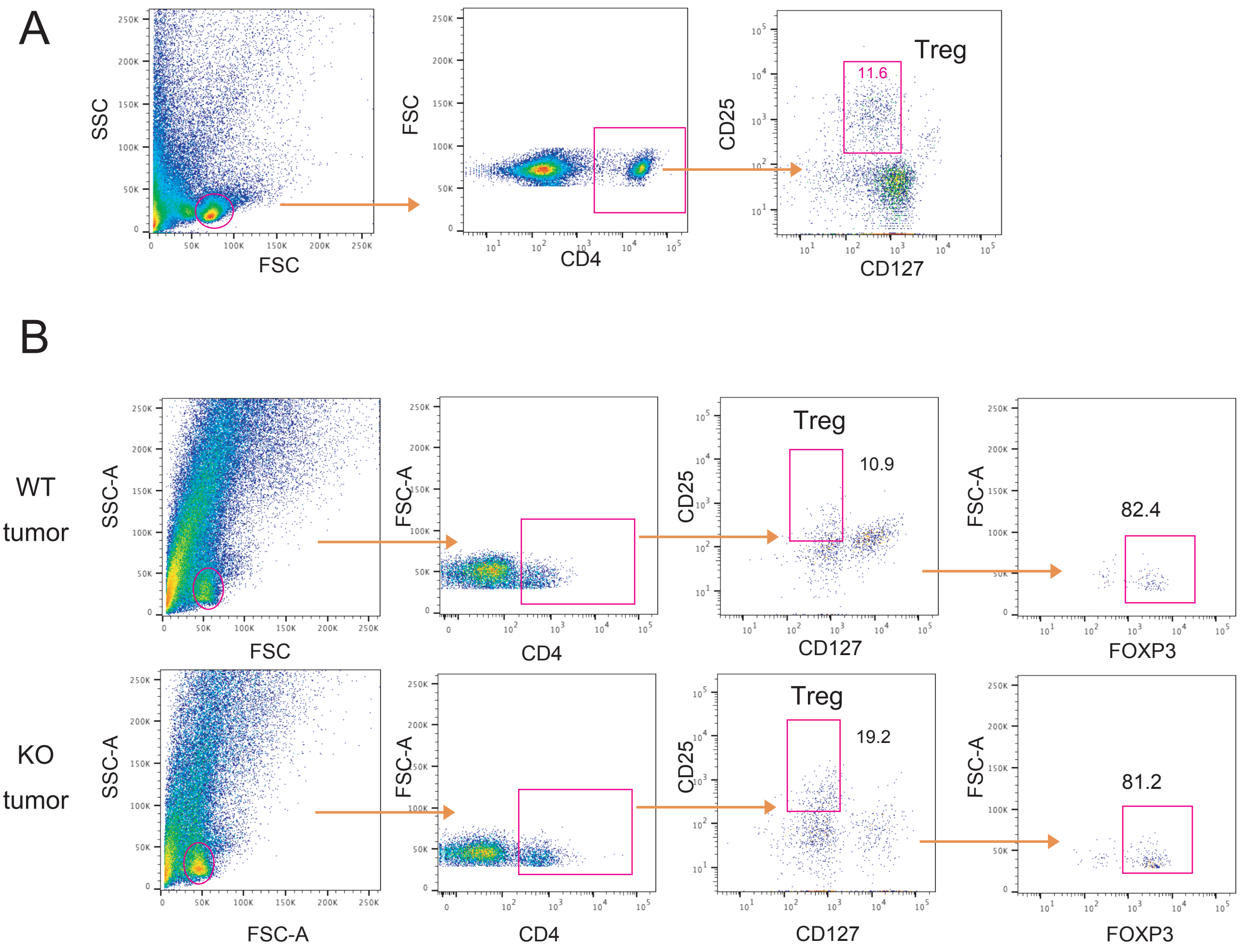

Supplementary Figure 3 CD4$^+$ CD25$^+$ CD127^{lo} cells of inguinal LNs (A) and B16-F10 tumor in wild-type and *Ccl21a*-KO mice (B). Treg (CD4$^+$ CD25$^+$ CD127^{lo}) subset was distinguished by FSC, SSC, and CD4 profiles of tumor cells, and the percentage of FoxP3 was analyzed. Data shown are representative of two experimental repeats with three biological replicates.

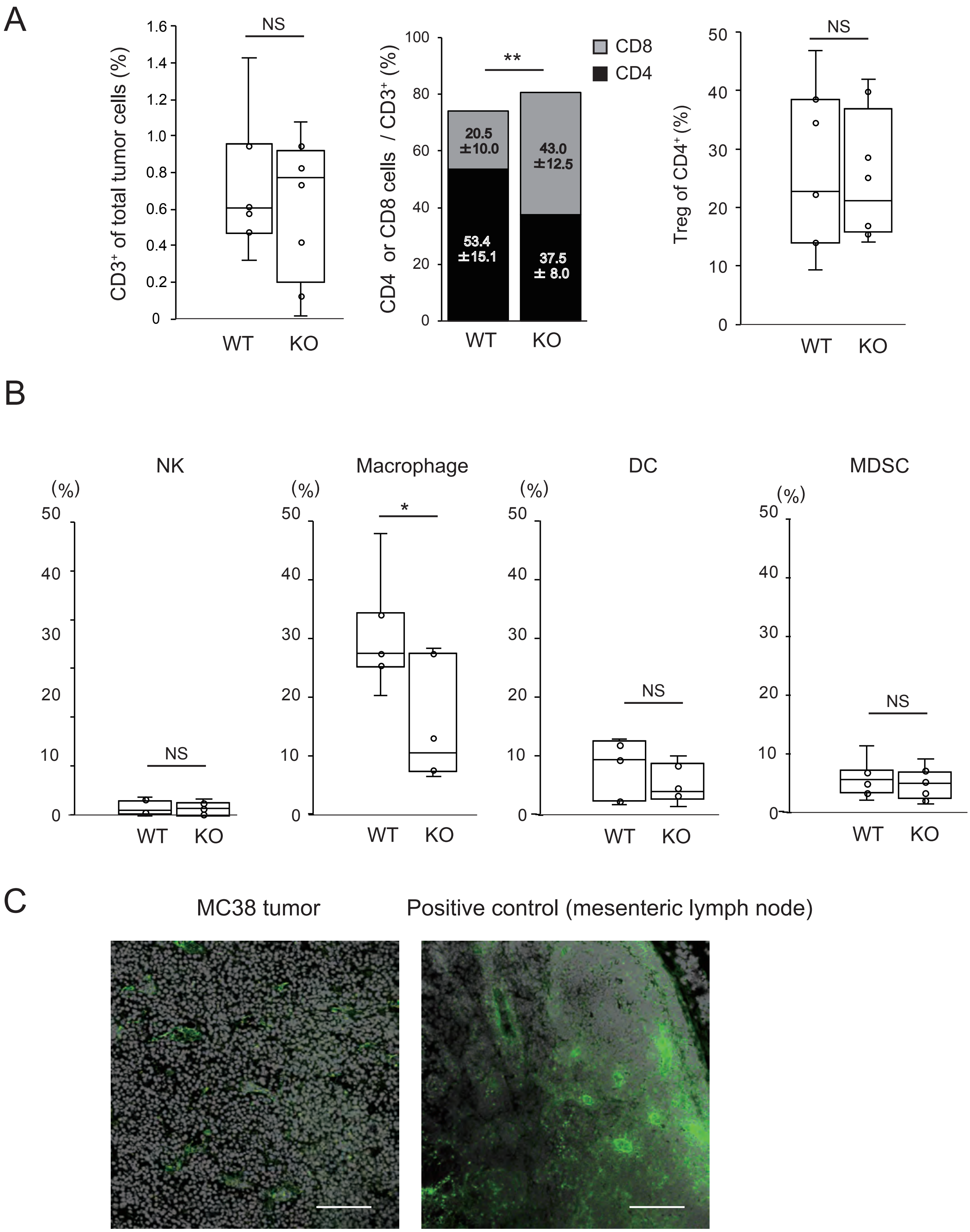

Supplementary Figure 4 IImmune cell infiltration in MC38 tumors. (A) The CD3 T cell pergentage, CD8/CD4 ratio, and Treg ($CD4^+CD25^+CD127^{low}$) percentage of CD4 T cells are shown. (B) NK cells ($NK1.1^+$ $CD3^-$ $CD69^+$), macrophage ($F4/80^+$), DCs ($F4/80^-$ MHC class II^+ $CD11c^+$) and MDSCs ($Gr\text{-}1^+$ $CD11b^+$) of $CD45^+$ cells in B16-F10 tumors were analyzed by flow cytometry.(C) Immunohistochemical analysis of MC38 tumor tissue. Green: CCL21, Gray: Hoechst33342. Scale bar: 100 μm
